# The Virus Entry Pathway Determines Sensitivity to the Antiviral Peptide TAT-I24

**DOI:** 10.3390/v17040458

**Published:** 2025-03-23

**Authors:** Eva Kicker, Antonio Kouros, Kurt Zatloukal, Hanna Harant

**Affiliations:** 1 Diagnostic and Research Center for Molecular Biomedicine, Diagnostic and Research Institute of Pathology, Medical University Graz, Neue Stiftingtalstraße 6, 8010 Graz, Austria; e.kicker@medunigraz.at (E.K.); antonio.kouros@medunigraz.at (A.K.); 2 Pivaris BioScience GmbH, Media Quarter Marx 3.4, Maria-Jacobi-Gasse 1, 1030 Vienna, Austria

**Keywords:** antiviral peptide, SARS-CoV-2, virus entry, endocytosis, Rab14

## Abstract

The peptide TAT-I24, a fusion of the TAT peptide (amino acids 48–60) and the 9-mer peptide I24, has been previously shown to neutralize several double-stranded (ds) DNA viruses in vitro. We have now extended the testing to potentially sensitive RNA viruses and analyzed the antiviral effect of the peptide against Severe Acute Respiratory Syndrome Coronavirus-2 (SARS-CoV-2). In Vero E6 cells, TAT-I24 neutralized the human 2019-nCoV isolate (Wuhan variant) in a dose-dependent manner, while it was unable to neutralize two SARS-CoV-2 variants of concern, Delta and Omicron. Moreover, TAT-I24 could not significantly neutralize any of the SARS-CoV-2 variants in the human lung carcinoma cell line Calu-3, which provides an alternative entry route for SARS-CoV-2 by direct membrane fusion. Therefore, a possible dependence on virus uptake by endocytosis was investigated by exposing Vero E6 cells to chloroquine (CQ), an inhibitor of endosomal acidification. The Wuhan variant was highly sensitive to inhibition by CQ, an effect which was further enhanced by TAT-I24, while the Delta variant was less sensitive to inhibition by higher concentrations of CQ compared to the Wuhan variant. The microscopic analysis of COS-7 cells using a rhodamine-labeled TAT-I24 (Rho-TAT-I24) showed the endosomal localization of fluorescent TAT-I24 and co-localization with transfected GFP-Rab14 but not GFP-Rab5. As these proteins are found in distinct endosomal pathways, our results indicate that the virus entry pathway determines sensitivity to the peptide.

## 1. Introduction

Viral infections are continuous challenges to the global health system. Viruses which can cross the species barrier from animals to humans are particularly dangerous, since they have not yet adapted to the human host. Global warming and the destruction of ecosystems by humankind affect wildlife populations and can lead to virus outbreaks, as recently experienced with the outbreak of SARS-CoV-2. This virus started to emerge in 2019 and led to a pandemic with millions of deaths. As the health care systems were not prepared for this type of virus, suitable vaccines or antiviral drugs were not available at the onset of the pandemic and so countries enforced social distancing measures by lockdowns [1,2].

In an unprecedented global effort, vaccines were developed, based on either inactivated viruses, genetically modified adenoviruses, or newly developed mRNA vaccines. In addition, antibodies, as well as repurposed and new drugs, have been developed [3]. However, mutations led to new virus variants which could escape these treatments [4,5,6]. Therefore, there is a high unmet medical need to develop novel, broad-acting antivirals. Such agents could either target the host cell or a common viral target, including the viral genome or viral polymerases, but also the entry path into the host cell. However, due to the high diversity of viruses, common targets are difficult to identify [7,8,9,10].

Apart from small molecules, peptides are developed for antiviral treatment [11,12], with two peptide entry inhibitors already on the market: Fuzeon^®^ (enfuvirtide; Roche, Basel, Switzerland) for the treatment of human immunodeficiency virus-1 (HIV-1) infections [13] and Hepcludex^®^ (bulevirtide; Gilead Sciences, Foster City, CA, USA) for the treatment of chronic hepatitis D infections [14]. Several other peptides have been identified which can inhibit viral entry into the host cells by targeting viral fusion with the host cell membrane [15,16,17,18]. Other peptides target the viral envelope, such as LL-37 [19,20], or endosomal acidification, such as P9R [21].

We have previously identified the peptide TAT-I24, a fusion of the well-known cell-penetrating TAT peptide (amino acid residues (aa) 48–60) [22,23,24] and the 9-mer peptide I24, which can neutralize a variety of dsDNA viruses in vitro, including herpes simplex viruses, cytomegalovirus and vaccinia virus. It also neutralizes some adenoviruses and SV40 polyomavirus, which are both non-enveloped dsDNA viruses [25]. The peptide acts at the early stages of viral entry through binding to the cell membrane, followed by internalization, a process mediated by the cationic TAT peptide [25]. Additionally, TAT-I24 has a strong binding affinity for dsDNA, which is about 100-fold higher compared to the TAT fusion partner [26]. The proposed mechanism of action therefore involves the co-internalization of the peptide with the virus and the binding of the peptide to the viral DNA upon uncoating, leading to the inhibition of further events, such as viral gene transcription [25,26]. If this also holds true for viral RNA, this could be a mechanism which is potentially applicable to a wide range of viruses.

In previous studies, we have shown that TAT-I24 exerted partial antiviral effects against the retrovirus HIV-1 as well as against the RNA virus respiratory syncytial virus (RSV). In contrast, the peptide was inactive against the RNA virus influenza A in MDCK cells [25]. Since many pathogenic viruses contain RNA genomes, one open question is which RNA viruses can be neutralized by TAT-I24. Although our earlier results with RNA viruses indicated only partial or no sensitivity to TAT-I24, the selection of the investigated viruses was too small to conclude that RNA viruses would be generally insensitive to TAT-I24. Therefore, the aim of the present study was to analyze whether and how TAT-I24 interferes with SARS-CoV-2 infections.

## 2. Materials and Methods

### 2.1. Peptides and Compounds

The peptide TAT-I24 (GRKKRRQRRRPPQCLAFYACFC) was synthesized at Bachem AG (Bubendorf, Switzerland). TAT (aa 48–60; GRKKRRQRRRPPQ) and the TAT-I24 analog C14A, C20R, C22M [27] were synthesized at ProteoGenix (Schiltigheim, France). The TAT-I24 analogs C14P, C22del, and C22del_cyc [27], the scrambled peptides 1 (GACPQLRRCRYKKFFRRACQRP) and 2 (CCPPRLRQFRGCFRAYAQRRKK), as well as the amino-terminal rhodamine-labeled TAT-I24 (Rho-TAT-I24) were synthesized at Synpeptide (Shanghai, China). All peptides were purified to >95%. The peptides were dissolved in DMSO as 10 mM stock and stored at −20 °C.

Remdesivir (#HY-104077) was purchased from MedChemExpress (Vienna, Austria). Chloroquine diphosphate (CQ; #C6628) was purchased from Merck (Schnelldorf, Germany). A 10 mM stock was made in distilled water, filtrated using a 0.2 µm filter, and stored at 4 °C for a maximum of three months. Dilutions for use in infection assays were made in phosphate-buffered saline (PBS) and freshly prepared prior to the experiments.

### 2.2. Viruses

All experimental steps involving infectious SARS-CoV-2 were performed in a BSL-3 laboratory.

#### 2.2.1. SARS-CoV-2

The human 2019-nCoV isolate (Ref-SKU: 026V-03883; strain BavPat1/2020 further designated as the Wuhan variant) from the European Virus Archive-Global (EVAg; Marseille, France) was propagated in Vero E6 cells, and titers were determined by counting the plaque forming units 72 h after infection with the serially diluted virus, as described in Hardt et al. [28]. The Delta variant was isolated from a patient sample at the Medical University Graz and was confirmed by sequencing (sequence information: www.gisaid.org (accessed on 6 October 2024) [29]; Accession ID: EPI_ISL_4847176) and propagated using the same procedure as above. The Omicron variant, lineage B1.1.529, was purchased from EVAg (Ref-SKU: 010V-04425). All virus stocks were generated with the same method as described above. For all infection experiments, the working stocks were diluted to a calculated multiplicity of infection (MOI) of 0.002, if not stated otherwise [28].

#### 2.2.2. Baculovirus

A baculovirus expressing firefly luciferase (baculovirus-Luc), kindly provided by Reingard Grabherr, Institute of Molecular Biotechnology, BOKU University, Vienna, Austria, has been described previously [25,26,27].

### 2.3. Cell Culture

Vero E6 cells (VC-FTV6) were obtained from Vircell S.L. (Granada, Spain) via Biomedica (Vienna, Austria). The cells were cultured in Gibco’s Minimal Essential Medium (MEM) containing Earle’s Salts and L-glutamine, and supplemented with 5% fetal calf serum (FCS) and 1% penicillin + streptomycin (Thermofisher, Darmstadt, Germany). The assay medium using Vero E6 cells was MEM, containing the above listed additives, except with only 2% FCS used.

The cell line Calu-3 was obtained from the cell bank of the Medical University Graz (Centre for Medical Research, Graz, Austria) and cultured in MEM containing Earle’s Salts and L-Glutamine, and supplemented with 10% FCS and 1% penicillin + streptomycin (Thermofisher, Darmstadt, Germany). Both cell lines were maintained at 37 °C in a humidified atmosphere containing 5% carbon dioxide (CO_2_).

COS-7 cells were grown in CO_2_-independent medium supplemented with 10% FCS, 2 mM glutamine, and 1% antibiotic–antimycotic (ThermoFisher, Darmstadt, Germany) and cultivated in a humidified atmosphere at 37 °C. The cells were passaged once a week.

### 2.4. Virus Neutralization Assay and Quantification of Virus RNA in the Supernatants

For the infection experiments, Vero E6 cells were seeded 24 h (24 h) prior to the assay in a 48-well plate (Corning Costar, Corning, NY, USA) in MEM containing 2% FCS at a density of 3.0 × 10^4^ cells per well. Calu-3 cells were seeded at a density of 4 × 10^4^ cells per well in MEM containing 10% FCS.

On the day of infection, the seeding medium was discarded, and the cells were exposed to different peptide or compound concentrations, diluted in MEM containing 2% FCS, and subsequently infected with SARS-CoV-2 (MOI 0.002). After 1 h, 140 µL of supernatant (=t0) was harvested and frozen for later analysis. Infected cells were maintained at 37 °C and 5% CO_2_ for 24 h. On the following day, 140 μL supernatants were harvested and inactivated by the addition of viral lysis buffer. RNA extraction was performed using QIAmp Viral RNA Mini Kit (Qiagen, Hilden, Germany) according to the manufacturer’s instructions.

Viral RNA was amplified with a Rotor Gene Q cycler (Qiagen, Hilden, Germany) and the QuantiTect Multiplex RT-PCR Kit (Qiagen, Hilden, Germany) combined with the recommended primers and probe set N2 from CDC (2019-Novel Coronavirus (2019-nCoV) Real-time rRT-qPCR Panel) [30]. The reaction volume was reduced to 25 μL, and with the abovementioned QuantiTect Multiplex RT-PCR Kit, reverse transcription was performed for 30 min (min) at 50 °C, followed with the initial activation of the polymerase for 15 min at 95 °C. The 2-step cycling profile consisted of 45 amplification cycles with denaturation at 95 °C and annealing at 55 °C.

### 2.5. Detection of Viral RNA from Cell Lysates

Infection experiments were performed as described above, with the exception that the infected cells were lysed after the indicated time points. For this, the supernatants were removed carefully, and the cells were washed three times with PBS and then lysed with RLT buffer (supplemented with 1% ß-mercaptoethanol) from the RNeasy Micro+ Kit (Qiagen, Hilden, Germany). The lysates were processed following the manufacturers protocol and eluted in 14 µL of RNAse-free H_2_O. RNA concentrations were quantified using a Nanodrop 1000 spectrometer. A cDNA synthesis was performed from 100 ng total RNA using the NEB LunaScript^®^ RT-SuperMix Kit (#E3010L, New England BioLabs, Frankfurt am Main, Germany). Before PCR, cDNA was diluted 1:3 in H_2_O. For the detection of subgenomic and genomic viral RNA, primers were synthesized according to the primer sequences in the publication by Telwatte et al. [31] at Microsynth AG (Balgach, Switzerland). Relative transcript levels, normalized to the housekeeping gene GAPDH, were calculated using the 2^−ΔΔCT^ method [32]. For the amplification of GAPDH mRNA, the primers GAPDH forward, 5′-GTCAGCCGCGTCTTCTCTT-3′, and GAPDH reverse, 5′-GCGCCCAATACGACCAAATC-3′, were used. Quantitative RT-PCR was performed using the Luna^®^ Universal qPCR Master Mix from NEB (#M3003E, New England BioLabs, Frankfurt am Main, Germany) in 96-well plates using a QuantStudio Real-time PCR system (QuantStudio Flex 6; Applied Biosystems/Thermo Fisher Scientific Inc.; Waltham, MA, USA) and corresponding software for the analysis of the results.

### 2.6. Immunohistochemical Staining

Immunohistochemistry was conducted following previously established protocols [33]. Infected cells from the experiments described in Section 2.4. were fixed with 4% buffered formaldehyde for 30 min and then permeabilized using 0.1% Triton X-100 in PBS for a duration of 10 min. The activity of endogenous peroxidases was blocked by treating the cells with 3% H_2_O_2_ in methanol for 30 min. Subsequently, the cells were washed three times with PBS and incubated for 1 h with a primary antibody (SARS-CoV-2 (2019-nCoV) Nucleocapsid Antibody, Rabbit Mab #40143-R019) from Sino Biological (Beijing, China) at a 1:1000 dilution in antibody diluent (#S202230-2, REAL Antibody diluent) from Agilent Technologies (Vienna, Austria). Following the primary antibody incubation, the cells were treated with the secondary antibody (EnVision™ + Dual Link System HRP, #K5007; Agilent Technologies; Vienna, Austria). After three additional washes with PBS, the cells were exposed to the DAB substrate Chromogen (#K5007; Agilent Technologies; Vienna, Austria) and incubated until the virus-infected cells were stained red, for a maximum of 3 min. The reaction was stopped by washing the wells three times with PBS, and afterwards the cells were maintained in a humid environment. For image documentation, a Nikon Eclipse TS100 microscope (Nikon, Tokyo, Japan) with two-fold or four-fold magnification, along with a JENOPTIK GRYPHAX^®^ camera and software (Jena, Germany), were used.

### 2.7. Immunofluorescence

For the immunofluorescence staining assays, Vero E6 were cultured in 24- and 12-well plates (Corning Costar; Corning, NY, USA), with sterile glass coverslips placed inside the wells. The coverslips were treated with 0.1% gelatine and dried under sterile conditions. After that, Vero E6 cells were seeded with a density of 4 × 10^4^ or 4 × 10^5^ cells per 24- or 12-well, respectively. After incubation for 24 h, the cells were infected with the virus and incubated for the indicated time points. After infection, the cells were washed three times with PBS and fixed with 4% buffered formaldehyde for 30 min. After three more washing steps in PBS, the cells were permeabilized with Triton X-100 (diluted to 0.1% in PBS) for 10 min, followed by further washing and blocking in 3% BSA (Bovine Serum Albumin) in PBS + 2% goat serum (Merck; #G9023, Schnelldorf, Germany). The slides were then incubated with the primary antibody, diluted 1:1000 in 1% BSA overnight at 4 °C, which was mouse mAb anti-dsRNA J2 (Jena Bioscience, Jena, Germany; #RNT-SCI-10010200). After washing three times, a secondary fluorescent-labeled antibody (Alexa Fluor 488 goat-anti mouse; #A-11008, Invitrogen/Thermofisher; Waltham, MA, USA) was diluted 1:400 in 1% BSA and the coverslips were incubated in the dark for 1 h. After washing, the coverslips were carefully lifted from the cell culture plates, flipped in reverse, and placed on a glass slide spotted with 20 µL of ProLong^®^ Gold Antifade Mountant with DNA stain 4′,6-Diamidino-2-Phenylindole, Dihydrochloride (DAPI) (#P36931, Invitrogen/Thermofisher, Waltham, MA, USA;). The coverslip and glass slide were dried overnight in a dark environment and sealed on the following day using commercially available nail polish. Fluorescent images were acquired using OLYMPUS IX83 microscope (Tokyo, Japan) with the corresponding software OLYMPUS cellSens Dimension 4.2.1.

### 2.8. Luciferase Reporter Gene Assays

The transduction of COS-7 cells with baculovirus-Luc was performed as described previously [26]. The luciferase levels were determined 24 h after transduction.

### 2.9. RNA Binding Assay

A DNA fragment was generated by conventional PCR from pcDNA^TM^3.1(+) (Invitrogen/Thermofisher; Waltham, MA, USA) using the forward primer 5’-GAAATTAATACGACTCACTATAGGG-3’ and the reverse primer 5’-ATTAGGAAAGGACAGTGGGA-3’, yielding a 263-base-pair PCR product. The primers were synthesized at Microsynth AG (Balgach, Switzerland). The fragment contained the T7 promoter and was used as template for RNA synthesis by T7 polymerase using the HiScribe^®^ T7 High Yield RNA Synthesis Kit (#E2040S, New England BioLabs, Frankfurt am Main, Germany), conducted overnight at 37 °C. The RNA was treated with RNAse-free DNase I (Qiagen, Hilden, Germany), extracted with phenol/chloroform/isoamylalcohol (25:24:1), and then precipitated with 1/10 volume of 3M sodium acetate and 2.5 × volume of 96% ethanol, followed by centrifugation for 10 min in a microcentrifuge and washing with 80% ethanol. The pellet was then dried and dissolved in nuclease-free water. The RNA concentration was determined by measuring the optical density at 260/280 nm. The RNA (1 µg/well) was then incubated with increasing concentrations of peptides in 50 µL of TE buffer in black 96-well plates. After 5 min, 5 µL of SYBR^®^ Gold stain (ThermoFisher, Darmstadt, Germany) was added (diluted 1:400 from concentrate) and fluorescence was recorded using the GloMax^®^-Multi Detection System (Promega, Mannheim, Germany) and the optical kit blue (excitation wavelength 490 nm, emission wavelength 510–570 nm).

### 2.10. Fluorescence Microscopy of COS-7 and Vero E6 Cells Treated with Rho-TAT-I24

COS-7 cells or Vero E6 cells were seeded at a density of 2 × 10^4^ cells/250 µL per well in ibiTreat 8-well chambers (ibidi GmbH, Gräfelfing, Germany) in a CO_2_-independent medium supplemented with 10% FCS and allowed to attach overnight at 37 °C. The rhodamine-labeled peptide Rho-TAT-I24 was diluted 1:50 in PBS before the addition of 950 µL of medium with 10% FCS. Then, the mixture was centrifuged for 5 min at 10,000× *g* to pellet the precipitate. The medium was removed from the cells and replaced by the medium containing Rho-TAT-I24 (10 µM) and further incubated at 37 °C for the indicated time points. After incubation, the cells were washed twice with PBS before fixation with 5% formaldehyde.

For the immunofluorescence staining of lysosomal-associated membrane protein 1 (LAMP1), the COS-7 cells were treated as above. After fixation, the cells were permeabilized with 0.5% Triton X-100 (Merck, Schnelldorf, Germany) in PBS for 10 min at room temperature. The cells were then incubated with 3% BSA (Merck, Schnelldorf, Germany) in PBS for one hour. After that, anti-LAMP1 antibody (#AB2971; Merck, Schnelldorf, Germany) was added at a dilution of 1:400 in 1% BSA in PBS overnight at 4 °C. After three washes, a secondary antibody (FITC-conjugated anti-rabbit IgG; #F0382; Merck, Schnelldorf, Germany) at a 1:100 dilution was applied in 1% BSA in PBS for one hour at room temperature. The cells were then washed three times and stained with DAPI, as described above.

Microscopy was performed with a 40 × oil objective using a Live Cell Video Microscope (Leica Microsystems, Wetzlar, Germany) provided by the BOKU Imaging Center (Vienna, Austria).

### 2.11. Plasmid Cloning and Transfection

The coding regions of Rab5 and Rab14 were amplified by PCR from the cDNA of human cells. Rab5 was cloned into the EcoRI and XbaI sites of pEGFP-C1 (Clontech, Mountain View, CA, USA), and Rab14 into the EcoRI site of pEGFP-C1. The correctness of the GFP-fusion constructs was confirmed by sequencing (Microsynth Austria GmbH, Vienna, Austria). For transfection, COS-7 cells were seeded at a density of 4 × 10^4^ cells/250 µL per well in ibiTreat 8-well chambers (ibidi, Gräfelfing, Germany) in a CO_2_-independent medium supplemented with 10% FCS and allowed to attach overnight at 37 °C. On the next day, the cells were transfected with pEGFP-C1-Rab5 or pEGFP-C1-Rab14 according to the following protocol: Transfection was performed with 2 µL of Lipofectamine 2000/well (Thermofisher, Darmstadt, Germany) and 0.8 µg of DNA/well in the medium without FCS. Five hours after transfection, the medium was replaced with medium containing 10% FCS. On the next day, the cells were incubated with Rho-TAT-I24 (10 µM) and transduced with baculovirus-Luc (MOI 30). After 120 min, the cells were washed three times, fixed with 5% buffered formaldehyde, and incubated with 115 µL of a solution of 1 µg/mL of DAPI (Invitrogen/ThermoFisher, Darmstadt, Germany) for 10 min before microscopy, as described in 2.10.

### 2.12. Statistical Analysis

Statistical analysis was performed using Graph Pad Prism 8 or 9 (GraphPad Software, San Diego, CA, USA).

### 2.13. Image Analysis

Images acquired after immunohistochemical or immunofluorescent staining were analyzed for positive areas and mean intensity signal proportions using ImageJ (Image Processing and Analysis in Java, version 1.54j 12 June 2024) freeware, developed by Wayne Rasband—NIH, University of Wisconsin, MI, USA. Measured intensity signals were normalized to positive signals from untreated, infected cell controls and compared to the other tested parameters. The co-localization of GFP-Rab proteins and Rho-TAT-I24 was determined using the color threshold tool in ImageJ.

## 3. Results and Discussion

### 3.1. TAT-I24 Neutralizes the SARS-CoV-2 Wuhan Variant in Vero E6 Cells

**Figure 1:**
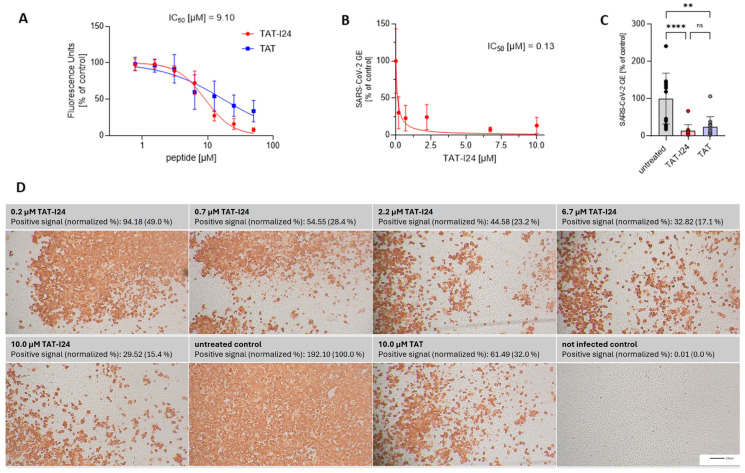
RNA binding of TAT-I24 or TAT and effect of peptides on replication of SARS-CoV-2 (Wuhan variant). (**A**) Binding of TAT or TAT-I24 to synthetic ssRNA. Mean ± SD of relative fluorescence units are shown (*n* = 6). (**B**) Relative viral genome equivalents (GE) in supernatants of Vero E6 cells infected with original Wuhan variant in presence of increasing concentrations of TAT-I24 (24 h p.i.). IC_50_ values were calculated by non-linear curve fit analysis. Data points show mean ± 95% confidence interval (CI) of *n* ≥ 9 from three independent experiments. (**C**) Relative SARS-CoV-2 GE in supernatants of Vero E6 cells infected with original Wuhan variant, untreated or treated with 10 µM of TAT-I24 or 10 µM of TAT for 24 h. Significant differences were detected using one-way ANOVA (Kruskal–Wallis multiple comparison of means). *p*-values: *p* ≥ 0.05 (ns); *p* ≤ 0.01 (**), and *p* ≤ 0.0001 (****); *n* = 12, mean ± SD are shown. (**D**) Corresponding wells (one representative experiment) from (B) with immunohistochemical staining against SARS-CoV-2 nucleocapsid. Images were quantified for red-positive area signals and normalized to untreated control (infected, untreated cells); representative sections of wells are shown. Scale bars indicate 200 µm.

TAT-I24 has been shown previously to bind DNA with a high affinity [26]. To further expand the testing to RNA, TAT-I24 was analyzed using an RNA-binding assay. Analogous to the DNA-binding assay used earlier [26], a single-stranded (ss) short synthetic RNA was generated and incubated with increasing concentrations of TAT-I24 or the fusion partner TAT in the presence of SYBR Gold dye. While the TAT peptide, which is known to bind nucleic acids [34,35,36], caused a dose-dependent reduction in fluorescence, the reduction by TAT-I24 was more pronounced (Figure 1A). However, the binding of TAT-I24 to RNA was less potent compared to its effect on dsDNA binding, with an IC_50_ of about 9 µM for RNA compared to an IC_50_ of approximately 1 µM for dsDNA [27].

SARS-CoV-2 is an ssRNA virus with positive polarity. In general, coronaviruses carry the largest RNA genomes, with that of SARS-CoV-2 consisting of about 30,000 nucleotides [37]. It was speculated that, because of the nucleic acid binding capacity of TAT-I24, viruses with large genomes may be more sensitive to inhibition by the peptide. We therefore investigated a potential antiviral effect of TAT-I24 against SARS-CoV-2. For this, Vero E6 cells were infected with the human 2019-nCoV isolate (Wuhan variant) in the presence of increasing concentrations of TAT-I24. Twenty-four hours post-infection (24 h p.i.), RNA was isolated from the cell culture supernatants. Quantitative reverse transcription polymerase chain reaction (qRT-PCR) was used to determine the viral copy numbers in the supernatants and the values were normalized to the untreated, infected control. As shown in Figure 1B, viral copy numbers in the supernatants were dose-dependently reduced by TAT-I24 with a calculated IC_50_ of 0.13 µM, which corresponds to previously calculated IC_50_ values with various viral systems [25]. At a single concentration of 10 µM, the TAT peptide also caused a reduction in viral copy numbers in the supernatants but was less effective compared to TAT-I24 at the same concentration (Figure 1C). A cell viability assay did not show any cell toxicity after 24 h by both peptides at concentrations up to 30 µM in Vero E6 cells (cytotoxicity concentration 50%, CC_50_ > 30 µM), as shown in Supplementary Figure S1. At 30 µM, TAT-I24 caused some morphological changes which were not associated with cytotoxicity (Supplementary Figure S2). This is in accordance with previous studies using DNA viruses which showed a sufficiently wide window between the antiviral effect and effects on cell viability [25,27]. In addition to the quantification of viral RNA in the supernatant, the infected cells were fixed with formaldehyde, and immunostaining was performed with an antibody detecting the viral nucleocapsid protein [33]. Red-positive scores from the immunostaining were quantified, with a clear, dose-dependent reduction in the number of positively stained cells by TAT-I24 being observed (Figure 1D), visually confirming the data shown in Figure 1B. Again, the fusion partner TAT, at a concentration of 10 µM, also caused a reduction in the percentage of positively stained cells (32% of untreated control), but less compared to TAT-I24 at the same concentration (15% compared to untreated control), as shown in Figure 1D.

**Figure 2:**
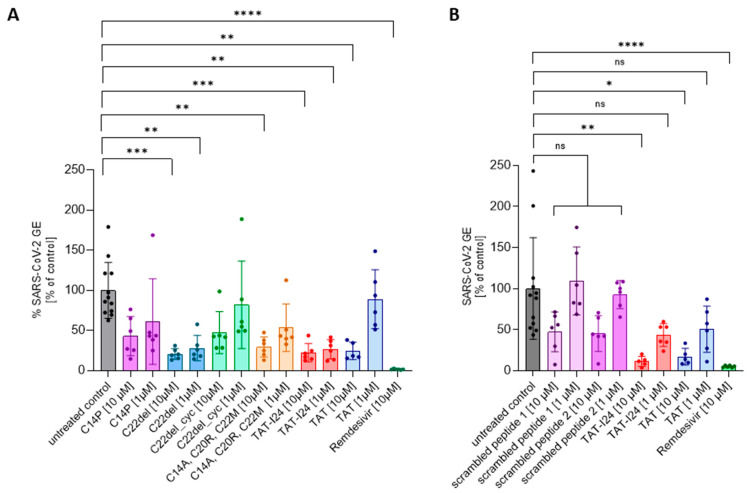
The effect of TAT-I24 analogs (**A**) and scrambled peptides (**B**) on the replication of SARS-CoV-2 (Wuhan variant). The relative viral genome equivalent (GE) in the supernatants of Vero E6 cells infected with the original Wuhan variant in the presence of 1 µM or 10 µM of TAT-analogs, TAT-I24, TAT, and scrambled peptides (24 h p.i.). The data points with the mean ± SD of *n* ≥ 6 from two independent experiments are shown. Significant differences were detected using one-way ANOVA (Kruskal–Wallis multiple comparison of means). *p*-values: *p* ≥ 0.05 (ns), *p* ≤ 0.05 (*), *p* ≤ 0.01 (**), *p* ≤ 0.001 (***), and *p* ≤ 0.0001 (****).

TAT-I24 contains three free cysteine residues which play a role in the antiviral effect, since the replacement of cysteines by other amino acid residues could alter antiviral potencies [27]. Lower sensitivity was observed for the variant C14P as well as for an analog with all three cysteines replaced (C14A, C20R, C22M). While the removal of C22 (C22del) in the linear peptide did not impact the antiviral effect, additional cyclization between C14 and C20 (C22del_cyc) greatly reduced its antiviral activity [27]. These analogs were therefore also tested at two concentrations (10 µM and 1 µM) against the SARS-CoV-2 Wuhan variant. As a reference, treatment with 10 µM of Remdesivir, an antiviral compound which potently inhibits the replication of SARS-CoV-2 [38], was used in all experiments. As with dsDNA viruses [27], variant C22del significantly reduced the SARS-CoV-2 virus copy numbers in the supernatants at 1 µM and 10 µM, while the variants C22del_cyc and C14P had no significant effect at both concentrations. The variant with all three cysteine replacements (C14A, C20R, C22M) could only significantly reduce the virus copy numbers at a concentration of 10 µM, which is in accordance with previous results showing activity against dsDNA viruses at higher concentrations [27]. The same was observed for the TAT peptide, which only caused significant inhibition at 10 µM, but not at 1 µM (Figure 2A). The effects of peptides on viral copy numbers in the supernatants were further confirmed by the nucleocapsid staining of infected cells (Supplementary Figure S3). These data demonstrate that similar features of the peptides are responsible for the antiviral effects against dsDNA viruses and SARS-CoV-2.

To further exclude any non-specific effect due to the general presence of peptides, two scrambled versions of TAT-I24 were generated and tested against the SARS-CoV-2 Wuhan variant. At 10 µM, both peptides had a partial, but not significant, inhibitory effect on the number of virus copies in the supernatants underlying the specific effect of TAT-I24 (Figure 2B), which was further confirmed by the nucleocapsid staining of infected cells (Supplementary Figure S4).

### 3.2. Sensitivity of SARS-CoV-2 to TAT-I24 Is Dependent on Virus Variant in Vero E6 Cells

During viral evolution, a variety of SARS-CoV-2 virus variant harboring mutations in the spike (S) protein appeared, and some of these variants continued to spread quickly all over the world. One such variant is the Delta variant, which was first detected in late 2020 in India, and which is characterized by several novel mutations within the S protein, including D614G, L452R, P681R, T478K, and D950N, enabling higher transmission rates compared to the original Wuhan isolate [39]. Ongoing viral evolution led to the emergence of the Omicron variant B.1.1.529, first appearing in 2021 and harboring additional mutations and replacing previous variants [40]. Although the Omicron variant is characterized by even higher infection rates, it causes less severe clinical symptoms and reduced mortality [41].

**Figure 3:**
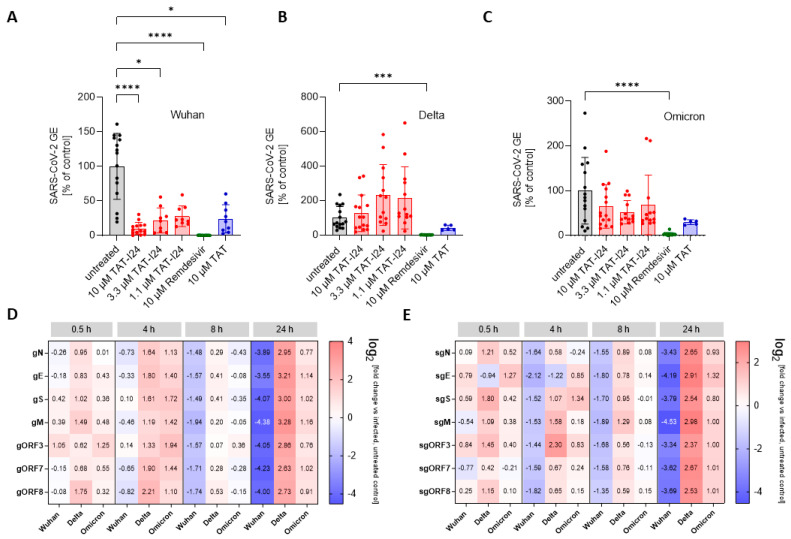
Differential inhibitory effect of TAT-I24 against SARS-CoV-2 virus variants. Relative viral GE in the supernatants of Vero E6 cells infected with the Wuhan variant (**A**), the Delta variant B.1.617.2 (**B**), and the Omicron variant B.1.1.529 (**C**) in untreated cells (gray), in the presence of various concentrations of TAT-I24 (red), 10 µM of Remdesivir (green), or 10 µM of TAT (blue) 24 h p.i.; significant differences were detected using one-way ANOVA (Kruskal–Wallis multiple comparison of means). *p*-values: *p* ≤ 0.05 (*); *p* ≤ 0.001 (***); and *p* ≤ 0.0001 (****), *n* ≥ 9, means ± SD from four independent experiments are shown for TAT-I24 with the Wuhan, Delta, and Omicron variants; for TAT, *n* ≥ 6 with the Wuhan, Delta, and Omicron variants from two independent experiments. Heatmap of the genomic (**D**) and subgenomic (**E**) SARS-CoV-2 RNA levels in the presence of 10 µM of TAT-I24 compared to untreated and infected controls; a 2 log-fold change at the time points 0.5 h, 4 h, 8 h, and 24 h p.i. for the Wuhan, Delta, and Omicron virus variants is shown.

To determine the sensitivity of these variants to TAT-I24, Vero E6 cells were infected with one Delta variant (B.1.617.2) and one Omicron variant (B.1.1.529) and treated with three different concentrations of TAT-I24 (10, 3.3, and 1.1 µM). The TAT peptide, at a concentration of 10 µM, was included for comparison. Remdesivir was used in all experiments as reference. As expected, TAT-I24 dose-dependently neutralized the SARS-CoV-2 Wuhan variant (9.9% GE of untreated control at 10 µM of TAT-I24, Figure 3A), albeit with a lower potency compared to Remdesivir (0.23% GE of untreated control). TAT also partially neutralized SARS-CoV-2 (24.4% GE of untreated control at 10 µM; Figure 3A), as already described above. Interestingly, the Delta variant could not be neutralized by any of the concentrations of TAT-I24 tested, while it remained fully sensitive to Remdesivir which inhibited to 0.62% GE of the untreated control (Figure 3B). It is possible that the Delta variant may have required higher concentrations of TAT-I24 compared to the Wuhan variant. However, at concentrations >30 µM, cell morphology is altered (Supplementary Figure S2); therefore, higher peptide doses were not applied. Surprisingly, at lower concentrations of TAT-I24 (3.3 and 1.1 µM), there was an increase in the viral copy numbers observed, albeit this effect was not statistically significant (Figure 3B). The Omicron variant did not significantly respond to TAT-I24 in either way, but was sensitive to Remdesivir, as shown in Figure 3C. Interestingly, the fusion partner TAT caused the partial inhibition of SARS-CoV-2 replication with all variants, with a reduction to 41.1% GE and 29.7% GE compared to the control for the Delta and Omicron variants, respectively (Figure 3B,C). In an earlier study using dsDNA viruses, a partial inhibitory effect of TAT was observed after the infection of NIH/3T3 cells with MCMV [25] and the antiviral effects of the TAT peptide have also been reported by other groups [42,43]. The cationic TAT peptide binds to negatively charged surface molecules, such as heparan sulfate proteoglycans [44,45,46]. These surface molecules are used by several viruses for cell attachment and have been reported to be involved in the cell binding of SARS-CoV-2 [47,48,49,50]. It is therefore likely that TAT reduces the overall cell attachment of SARS-CoV-2, which could explain the partial sensitivity of the three virus variants to the TAT peptide.

Since there can be considerable variabilities in viral copy numbers in cell culture supernatants, the effect of TAT-I24 on intracellular viral RNA levels was determined. For this, Vero E6 cells were infected with the SARS-CoV-2 Wuhan variant and the two SARS-CoV-2 variants, Delta and Omicron, in the absence and presence of 10 µM of TAT-I24, followed by cell lysis and the isolation of total cellular RNA at various time points, starting from 0.5 h until 24 h after infection. RNA was subjected to qRT-PCR using various primer pairs, detecting different segments of either genomic RNA (gRNA), which is indicative of active viral replication, or detecting various subgenomic RNAs (sgRNAs), which are indicative of viral gene expression [31]. The relative quantities of viral gene copies were calculated using the 2^−ΔΔCT^ method in relation to the GAPDH housekeeping gene from the peptide-treated cells to the untreated control [32]. As shown in Figure 3D, a reduction in genomic viral RNA levels was observed 8 h post-infection with the Wuhan variant in the presence of 10 µM of TAT-I24, with around 3-fold lower viral gRNA levels compared to untreated cells. This became further evident 24 h p.i., when the different viral gRNA levels were remarkably reduced (13- to 25-fold reduction compared to control). Interestingly, the enhancing effect of TAT-I24 on Delta virus copies in the supernatants was also observed in this setting, becoming most evident 24 h p.i. (6- to 9-fold higher gRNA levels), while the Omicron variant remained largely insensitive to the treatments (Figure 3D). A comparable trend was also observed at the levels of sgRNAs, which were also reduced when Vero E6 cells were infected with the Wuhan variant but enhanced when the cells were infected with the Delta variant in the presence of the peptide, with the effect being most visible 24 h p.i. (Figure 3E). However, regardless of the virus variants, similar changes in the levels of the different sgRNAs, derived from different SARS-CoV-2 genomic segments, were observed upon treatment with TAT-I24, i.e., inhibition for the Wuhan and enhancement for the Delta variant, indicating that the effect of TAT-I24 also occurs for SARS-CoV-2 at the level of virus entry or shortly thereafter. This is in accordance with an earlier report on the viral gene expression of MCMV-infected NIH/3T3 cells, where the transcript levels of both early and late genes were reduced by TAT-I24 to a similar extent [51].

**Figure 4:**
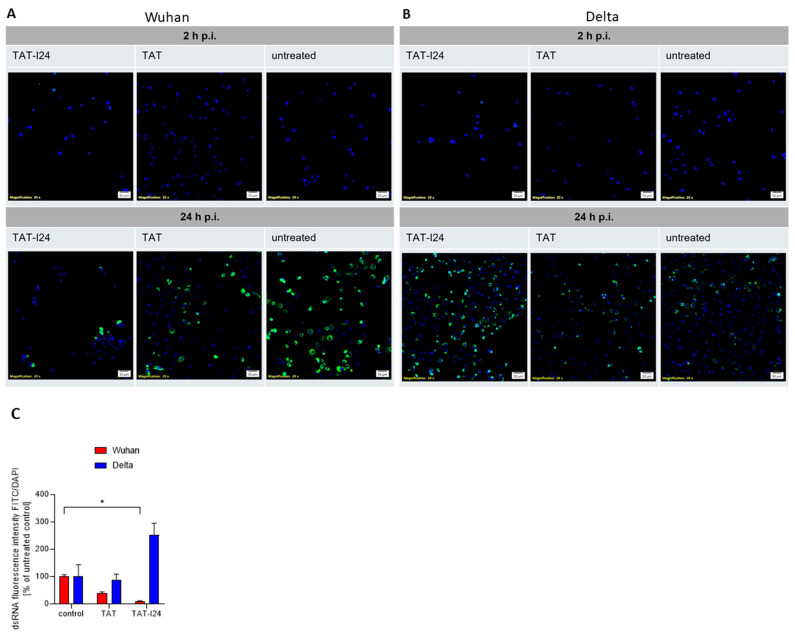
Effect of peptides on dsRNA levels in infected Vero E6 cells. (**A**) Cells were infected with Wuhan variant (500 pfu/well) or (**B**) Delta variant (500 pfu/well) for 2 h and 24 h in absence (untreated) and presence of TAT-I24 (10 µM) or TAT (10 µM), followed by staining for dsRNA (FITC/green); nuclei were stained with DAPI (blue), and one representative section is shown. Scale bars indicate 50 µm. (**C**) Quantification of dsRNA positive signals (FITC/green) from three randomly chosen sections normalized against DAPI (blue) signals and expressed as % of control; mean + SD from dsRNA relative to untreated controls are shown. Significant differences were detected using one-way ANOVA (Kruskal–Wallis multiple comparison of means); *p*-value: *p* ≤ 0.05 (*).

One hallmark of viral infections is the presence of dsRNA, an intermediate product of virus replication which plays a role in the induction of innate immune responses by SARS-CoV-2 [52,53]. We therefore studied the effect of TAT or TAT-I24 on the presence of dsRNA in Vero E6 cells infected with either the Wuhan variant or the Delta variant. Immunofluorescence staining using an antibody against dsRNA showed a reduction in fluorescent dsRNA signal in the presence of 10 µM of TAT-I24 or 10 µM of TAT after infection with the Wuhan variant (24 h p.i.) compared to untreated cells, while infection with the Delta variant and treatment with 10 µM of TAT-I24 also led to an enhanced effect on dsRNA signal, while a reducing effect was observed with 10 µM of TAT. As a control, the signal was evaluated at 2 h p.i. with no positive dsRNA staining observed, as active viral replication had not been initiated at this time point (Figure 4A,B) [54]. The intensity of the FITC signal from three randomly chosen areas was quantified and normalized to the corresponding DAPI signal from stained nuclei relative to the untreated control, as shown in Figure 4C.

### 3.3. Lack of Sensitivity to TAT-I24 in SARS-CoV-2-Infected Calu-3 Cells

It has been reported that there exist two different modes of SARS-CoV-2 entry, depending on the expression of surface molecules provided by different cell types [55]. Virus binding is mediated by the spike (S) protein of SARS-CoV-2 and the cellular receptor ACE2 (angiotensin-converting enzyme). Apart from the ACE2 receptor, several other co-factors such as heparan sulfate proteoglycans and other co-receptors, such as neuropilin, are involved [5,50,56,57,58]. Moreover, the cell-surface-located protease TMPRSS2 as well as the intracellular protease cathepsin L have been identified to be involved in the proteolytic processing of the S protein [54,59]. While SARS-CoV-2 entry occurs via endocytosis and requires endosomal acidification in cells lacking TMPRRS2, the infection of the human lung cancer cell line Calu-3, which expresses high levels of TMPRRS2, has been shown to be insensitive to chloroquine (CQ), an inhibitor of endosomal acidification, indicating a different entry route [54,60]. Moreover, the entry of SARS-CoV-2 in Calu-3 cells can be inhibited by camostat mesylate, an inhibitor of TMPRRS2, demonstrating that uptake in TMPRRS2-expressing cells occurs as a result of direct plasma membrane fusion bypassing endocytosis [54,60]. In Vero cells, which lack TMPRSS2, viral uptake is proposed to occur via pH-dependent endocytosis and transport to endolysosomes, with the activation of cathepsin L, while the overexpression of TMPRRS2 in Vero cells makes them insensitive to CQ, thus allowing pH-independent virus entry [60].

**Figure 5:**
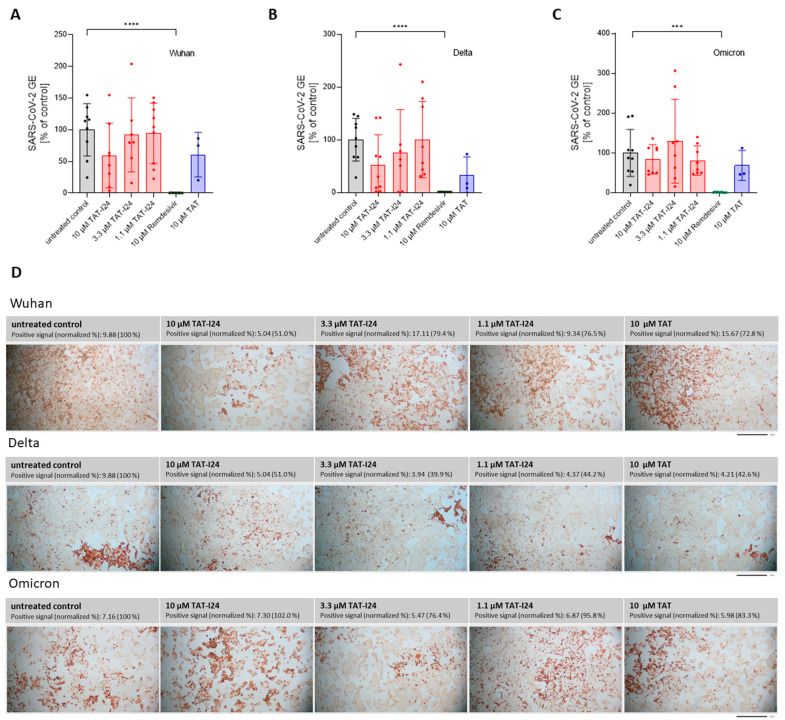
SARS-CoV-2 is not sensitive to TAT-I24 in Calu-3 cells. Relative viral GE in the supernatants of Calu-3 cells infected with the original Wuhan variant (**A**), the Delta variant B.1.617.2 (**B**), and the Omicron B.1.1.529 variant (**C**) in untreated cells (gray), in the presence of various concentrations of TAT-I24 (red), 10 µM of Remdesivir (green), or 10 µM of TAT (blue) 24 h p.i.; significant differences were detected using one-way ANOVA (Kruskal–Wallis multiple comparison of means). *p*-values: *p* ≤ 0.001 (***), *p* ≤ 0.0001 (****), *n* ≥ 6, the means ± SD from three independent experiments are shown for TAT-I24 and *n* = 3 for TAT from one experiment. (**D**) Staining of Calu-3 cells for SARS-CoV-2 nucleocapsid 24 h p.i. with the Wuhan variant, Delta variant, and Omicron variant. Images were quantified for positive area signals and normalized to the untreated control; representative sections of the wells are shown. Scale bars indicate 1 mm.

We therefore investigated whether infected Calu-3 cells with an uptake mechanism different to that of Vero cells were also sensitive to TAT-I24. As positive control, Remdesivir was also included, as it affects SARS-CoV-2 replication independent of cellular entry [38]. In contrast to Vero E6 cells, the SARS-CoV-2 Wuhan variant was largely insensitive to TAT-I24 upon the infection of Calu-3 cells, with only a partial neutralization observed at 10 µM of TAT-I24 (59.5% GE compared to control), the highest concentration tested. This extent of inhibition was similar to the effect caused by 10 µM of TAT (60.7% GE compared to control), as shown in Figure 5A. When Calu-3 cells were infected with the SARS-CoV-2 Delta variant, partial inhibition was observed, both with TAT (32.7% GE compared to control) and TAT-I24 (52.8% GE compared to control), at 10 µM, while no enhancing effect on viral copy numbers, as seen with Vero E6 cells, was observed for Calu-3 cells (Figure 5B). The Omicron variant did not respond to either TAT or TAT-I24 (Figure 5C). A significant reduction in virus copies in the supernatants was only observed by 10 µM of Remdesivir (0.48%, 1.1%, and 1.2% GE compared to the control for the Wuhan variant, Delta variant, and Omicron variant, respectively), confirming the validity of these experiments (Figure 5A–C). The qRT-PCR data obtained for the virus copies in the supernatants were also reflected at the level of the cells stained with an antibody against the viral nucleocapsid protein (Figure 5D). These data indicated that the different entry modes of SARS-CoV-2 in Calu-3 cells may have been responsible for the insensitivity to TAT-I24, and that virus uptake via endocytosis may be a prerequisite for inhibition by TAT-I24.

### 3.4. Differential Sensitivities of SARS-CoV-2 Wuhan and Delta Variants to CQ

**Figure 6:**
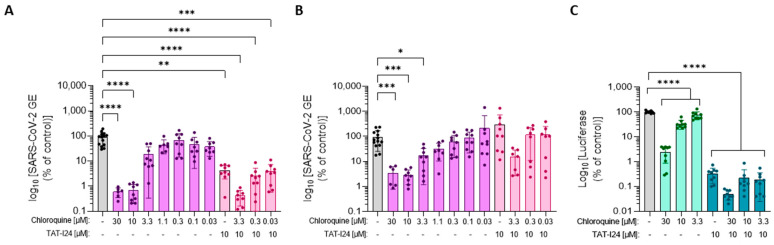
Differential sensitivity of the Wuhan variant and Delta variant to high concentrations of CQ. Vero E6 cells were infected with the SARS-CoV-2 Wuhan variant (**A**) or the Delta variant (**B**) in the presence of increasing concentrations of CQ (magenta) or combinations of CQ with 10 µM of TAT-I24 (pink). The mean ± SD of GE 24 h p.i. relative to the untreated control is shown. Significant differences were detected using one-way ANOVA (Kruskal–Wallis multiple comparison of means) from three independent experiments. *p*-values: *p* ≤ 0.05 (*); *p* ≤ 0.01 (**); *p* ≤ 0.001 (***); and *p* ≤ 0.0001 (****), *n* ≥ 6, mean ± SD are shown. (**C**) Luciferase levels in COS-7 cells infected with baculovirus-Luc in the presence of increasing concentrations of CQ (light green) and combinations of CQ with 10 µM of TAT-I24 (dark green). The data shown are the mean ± SD from the luciferase levels relative to the untreated control from three independent experiments (*n* = 9). Multiple *t*-test was used for statistical analysis; **** statistically significant at *p* ≤ 0.0001.

Since SARS-CoV-2 uptake occurs via the endolysosomal pathway in Vero cells, it was therefore questioned whether the different sensitivities of the original Wuhan variant and the Delta variant to TAT-I24 could be explained by differences in the mode of viral uptake by pH-dependent endocytosis in Vero E6 cells. To further analyze the entry pathway, Vero E6 cells were incubated with increasing concentrations of CQ and subsequently infected with SARS-CoV-2 Wuhan or Delta variants. In addition, combinations of various concentrations of CQ with 10 µM of TAT-I24 were tested. While the SARS-CoV-2 Wuhan variant was sensitive to inhibition by CQ (Figure 6A), the Delta variant was less sensitive at concentrations of 10 and 30 µM (Figure 6B). In addition, with the Wuhan variant, the combination of TAT-I24 with CQ further reduced the virus copies in the supernatant (Figure 6A), while no inhibitory effect of TAT-I24 when combined with any of the CQ concentrations was seen after infection with the Delta variant (Figure 6B). The corresponding images from the nucleocapsid staining are shown in Supplementary Figure S5.

This strongly suggests that the entry route of the Delta variant differs remarkably from the Wuhan variant in such a way that it becomes resistant to TAT-I24. It is well known that several of the S protein mutations in the Delta variant, including L452R and T478K, lead to an altered conformation of the receptor-binding domain (RBD) of the S protein and cause a different binding to the ACE2 receptor [61]. The D614G mutant has been reported to increase binding to ACE2, thereby enhancing viral entry [62]. The Delta variant also harbors another important mutation: the conversion of proline to arginine at position 681 (P681R). This mutation lies within the furin cleavage site, which is characteristic for SARS-CoV-2, and enhances the cleavage of the full-length spike to S1 and S2, associated with the enhanced viral transmissibility of the Delta variant [63,64,65]. It is possible that the altered receptor binding caused by the mutants in Delta affects the sensitivity to TAT-I24 by preventing the co-internalization of the virus with the peptide.

To study the dependence on endosomal acidification in other systems, a more convenient model was used, which did not require a specific safety environment, as working with SARS-CoV-2 does. This system employed an engineered baculovirus-expressing firefly luciferase which could transduce mammalian cells but was unable to replicate in these cells. A dose-dependent inhibition of reporter gene expression by TAT-I24 was observed after the transduction of COS-7 or HEK293 cells with this baculovirus [25,26,27]. When COS-7 cells were transduced with baculovirus in the presence of increasing concentrations of CQ, a dose-dependent inhibition of reporter gene expression was observed, indicating that the entry pathway of baculovirus in COS-7 cells involved pH-dependent endocytosis (Figure 6C). TAT-I24 inhibited luciferase expression in baculovirus-transduced cells also in the presence of CQ, and the combination of TAT-I24 with CQ at the highest concentration (30 µM) caused a further reduction in luciferase levels, demonstrating that TAT-I24 inhibited independently of endosomal acidification.

### 3.5. TAT-I24 Localizes to Endosomal Compartments

**Figure 7:**
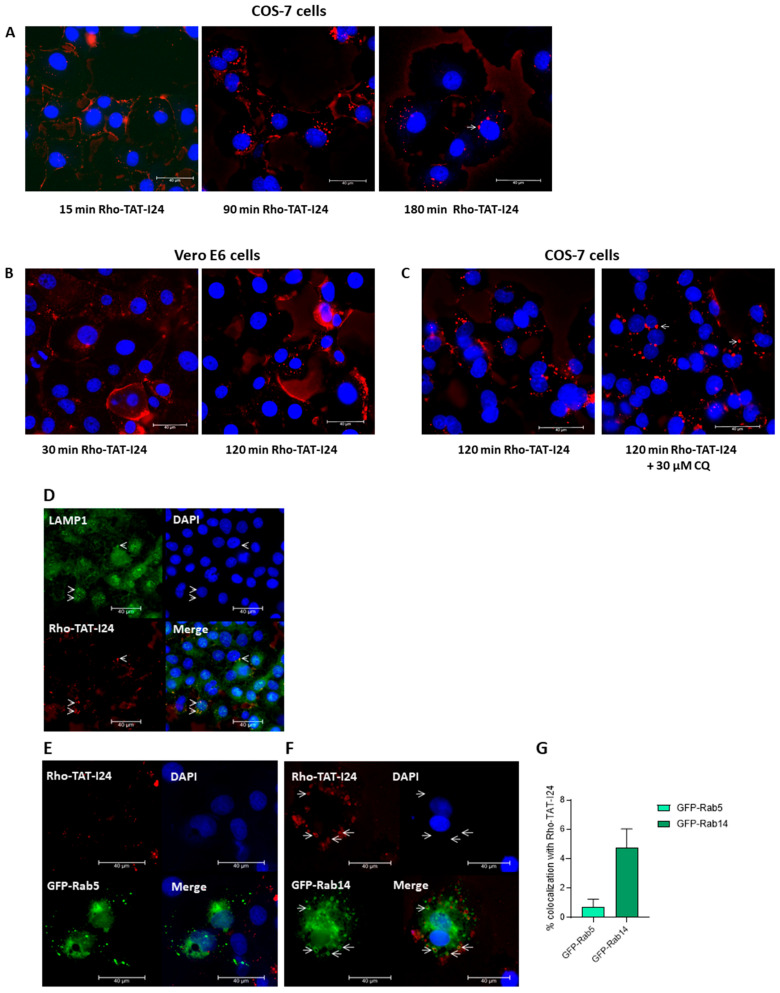
Localization of TAT-I24 in endosomal compartments. (**A**) Localization of Rho-TAT-I24 (red) in COS-7 cells after 15, 90, and 180 min incubation or (**B**) in Vero E6 cells after 30 and 120 min incubation. (**C**) Localization of Rho-TAT-I24 after 120 min either alone or in presence of 30 µM of CQ. (**D**) Staining of COS-7 cells for LAMP1 (green) after 30 min of incubation with Rho-TAT-I24 (red). Arrows indicate co-localization of Rho-TAT-I24 and LAMP1. (**E**) Overexpression of GFP-Rab5 (green) and lack of co-localization with Rho-TAT-I24 (red), or (**F**) overexpression of GFP-Rab14 (green) and incubation with Rho-TAT-I24 (red). Arrows indicate co-localization of GFP-Rab14 and Rho-TAT-I24 in enlarged vesicles. Nuclei are stained with DAPI (blue). Scale bars indicate 40 µm. (**G**) Co-localization of GFP-Rab5 or GFP-Rab14 with Rho-TAT-I24, expressed as percent (%) of total GFP signal, was determined from four areas of two independent experiments using color threshold tool in ImageJ.

In a recent study, Trofimenko et al. reported that the uptake of polyamines or cationic peptides, such as TAT, occurs via an endosomal pathway depending on the Rab GTPase Rab14, while the Rab5-dependent pathway is not used by these molecules [66]. The Rab14-dependent pathway has been proposed to result in non-acidic, LAMP1-positive late endosomes. Moreover, classical inhibitors of endocytosis do not inhibit the uptake of cationic peptides [66]. To test whether TAT-I24 also uses this entry route, a rhodamine-labeled TAT-I24 (Rho-TAT-I24) was synthesized. The treatment of COS-7 cells showed that 15 min after treatment with 10 µM of Rho-TAT-I24, the peptide was located mainly at the cell membrane, while after 90 and 180 min, intracellular, fluorescent vesicles were found (Figure 7A). A similar pattern was observed with Vero E6 cells, with initial fluorescent staining of the cell membrane followed by intracellular red fluorescent vesicular structures (Figure 7B). In COS-7 cells, some of these fluorescent vesicles appeared enlarged, which was even enhanced by treatment for 120 min with 30 µM of CQ, an effect which has been previously reported to occur with endosomal vesicles upon treatment with CQ [67], as shown in Figure 7C. Rho-TAT-I24 fluorescence also co-localized with LAMP1 [68], confirming its presence in endosomal compartments as shown in Figure 7D.

To analyze whether TAT-I24 utilized the Rab14-dependent endosomal pathway, COS-7 cells were transfected with plasmid constructs encoding the fusion constructs of green fluorescent protein (GFP) and Rab5 (GFP-Rab5) or Rab14 (GFP-Rab14) and treated the next day with 10 µM of Rho-TAT-I24 and transduced with baculovirus for 120 min. However, while there was no obvious co-localization of GFP-Rab5 with Rho-TAT-I24 observed, a clear co-localization of Rho-TAT-I24 with GFP-Rab14 was seen (Figure 7E). This is in accordance with previously published data by Trofimenko et al. demonstrating the internalization of cationic peptides in a Rab14-dependent pathway [66]. In our experiments, the expression of GFP-Rab14 was found in enlarged vesicles. This observation has been previously described to occur with overexpressed Rab14 or to occur even more with dominant-negative mutants of Rab14, such as Q70L [69,70,71].

While the uptake of viruses by endocytosis has been extensively studied [72], less data are available on different endocytosis pathways. Rab14 has been shown to be involved in the endocytosis of the virus matrix protein VP40 of Ebola virus [73]. In addition, a dependence of virus uptake on Rab14 was reported for HIV-1 [74] and classical swine fever (CSF) virus [75]. Other viruses have also been reported to use alternative entry routes, and not the classical Rab5-dependent pathway, such as herpes simplex virus [76] or lymphocytic choriomeningitis virus (LCMV) [77,78].

It is therefore possible that TAT-I24 can only exert a virus-neutralizing effect when it is co-inserted with the virus in the same endosomal compartment, possibly via a Rab14-dependent pathway. However, the strong sensitivity of the Wuhan variant to CQ would argue against this model, as the Rab14-dependent pathway involves non-acidic compartments [66]. Alternatively, more than one pathway is used by the virus at the same time, with only one pathway being sensitive to the peptide, explaining the different IC_50_ values observed with various viral systems [25]. The observation that TAT-I24 further enhances the inhibitory effect of CQ on the Wuhan variant could argue for such a situation. The notion that the inhibitory effect of CQ in the peptide-insensitive Delta variant, which is also less sensitive to CQ, cannot be further affected by TAT-I24 could indicate that this variant uses a different entry route. Of note is the observation that TAT-I24, in contrast to the TAT peptide, even caused some enhancement of viral copy numbers when the cells were infected with the Delta variant. This could indicate that the blockade of a specific pathway by the peptide may even enhance uptake by another pathway and warrants further investigations.

A lack of sensitivity to TAT-I24 was observed earlier where various virus infection models were analyzed for the neutralizing effect of TAT-I24. For instance, no effect of TAT-I24 on influenza A was observed in infected MDCK cells. Moreover, while being active against adenovirus type 5 in HEK293 and A549 cells, TAT-I24 could not neutralize adenovirus type 4 and 19a/64 in A549 cells. It was already speculated that different receptor usages of insensitive viruses led to an uptake mechanism not targeted by TAT-I24 [25].

Although the TAT peptide also caused the partial neutralization of SARS-CoV-2 independent of the variant and cell line, the peptide TAT-I24 was unable to neutralize the Delta variant. This indicates that although TAT-I24 also contains the cationic TAT peptide, it behaves differently from the TAT fusion partner. It is assumed that TAT has a more unspecific effect by reducing virus attachment, while TAT-I24 has a more specific effect involving a distinct endosomal route. The dissection of these pathways to understand the conditions under which a virus can be neutralized by the peptide and how the peptide could be engineered to target an even broader spectrum of viruses is needed, and studies are currently underway. This should not only support the further development of this peptide, but also makes TAT-I24 an interesting tool to investigate modes of viral entry.

## 4. Patents

Hanna Harant is the inventor of patent application WO2019/057973 “Gene expression inhibitors”.

## Data Availability

Raw and processed data supporting the results from this study can be shared after request to E.K. or H.H.

## Supplementary Materials

The following supporting information can be downloaded at: https://www.mdpi.com/article/10.3390/v17040458/s1, S1: Relative metabolic activity in Vero E6 and Calu-3 cells in the presence of TAT-I24 or TAT after 24 h; Figure S1: Relative metabolic activity in Vero E6 and Calu-3 cells in the presence of peptides; S2: Morphology of Vero E6 and Calu-3 cells after treatment with TAT-I24 or TAT after 24 h; Figure S2: CV staining in Vero E6 and Calu-3 cells in the presence of peptides; S3: SARS-CoV-2 nucleocapsid staining of Vero E6 cells treated with TAT-I24, TAT, or TAT-I24 analogs 24 h p.i. with the Wuhan variant; Figure S3: Immunohistochemical staining against the SARS-CoV-2 nucleocapsid in Vero E6 cells infected with the Wuhan variant in the presence of peptides; S4: SARS-CoV-2 nucleocapsid staining of Vero E6 cells treated with TAT-I24, TAT, or scrambled peptides 24 h p.i. with the Wuhan variant; Figure S4: Immunohistochemical staining against the SARS-CoV-2 nucleocapsid in Vero E6 cells infected with the Wuhan variant in the presence of peptides; S5: SARS-CoV-2 nucleocapsid staining of Vero E6 cells treated with CQ, TAT-I24, and a combination of the compounds 24 h p.i. with the Wuhan variant and the Delta variant; Figure S5: Immunohistochemical staining against the SARS-CoV-2 nucleocapsid in Vero E6 cells infected with the Wuhan variant (A) or the Delta variant (B).
