# Supplementary material for: The Virus Entry Pathway Determines Sensitivity to the Antiviral Peptide TAT-I24
Source: Viruses. 2025 Mar 23;17(4):458. doi: 10.3390/v17040458 (PMC12031635; doi:10.3390/v17040458)
Supplement: Supplementary file 1 [file viruses-17-00458-s001.zip › viruses-3551604-supplementary.pdf]

Supplementary data:

S1: Relative metabolic activity in Vero E6 and Calu-3 cells in the presence of TAT-I24 or TAT after 24 h

Method:

For the metabolic activity (MA) assays, Vero E6 cells or Calu-3 cells were seeded in 48-well cell culture plates at a density of 3.0E+04 and 4.0E+04 cells per well, respectively, and incubated in assay medium MEM with 2% FCS (Vero E6) and MEM with 10% FCS (Calu-3). After 24 h (for Vero E6) or 48 h (for Calu-3), the medium was replaced by medium containing peptide dilutions, with 0.1 % DMSO as vehicle control or with the medium alone (untreated). All incubations were performed in triplicate. After 24 h incubation in the presence of peptides, resazurin (10 µM) was added and the increase in relative fluorescent units (RFU) was measured for 160 min at a wavelength of 485/520 nm. From the obtained data points, linear regression analysis was performed. The slopes from peptide-treated wells were normalized to those of the untreated controls to calculate the relative metabolic activity in percent.

Results:

TAT-I24 has no cytotoxic effect on Vero E6 and Calu-3 cells

To exclude the cytotoxic effects of the peptide, a metabolic activity (MA) test was conducted. In the presence of metabolically active cells (NADH/H+ to NAD+/H2O), resazurin was reduced to resorufin. The fluorescence signal was directly proportional to the amount of NAD+ released by intact, metabolically active cells and was measured at 485/590 nm over a period of 160 min.

The medium with or without peptide dilutions was incubated in the absence of cells for the same duration and measured in parallel. These values were subtracted as respective blanks. Regression lines were calculated from the measured values, and the slope (k) was compared. The slope (k) was normalized to the untreated control, representing the relative metabolic activity (RMA %).

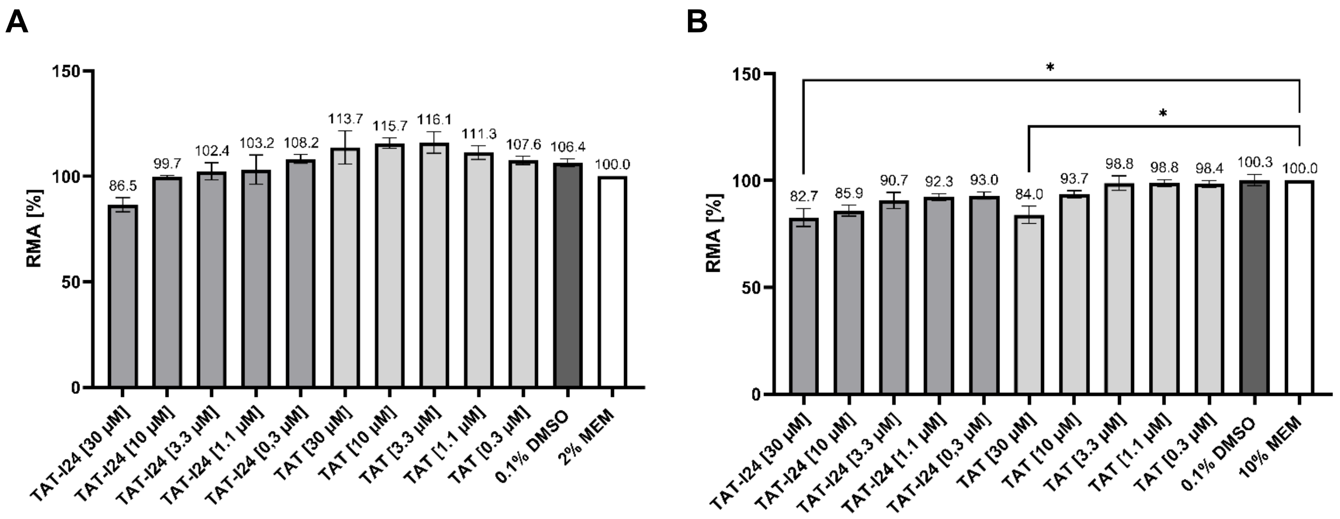

Figure S1: Relative metabolic activity in Vero E6 and Calu-3 cells in the presence of peptides. The RMA (%) of Vero E6 cells (A) and Calu-3 cells (B) after 24 hrs of incubation with peptides is shown. Statistically significant differences in the means from the absolute slopes were calculated using one-way ANOVA (Kruskal–Wallis multiple comparison of means). P-values: $p \leq 0.05$ (*); the results shown are the mean ± SD in percent compared to the untreated control from triplicate analyses.

As shown in Fig. S1, no significant differences in RMA were seen after 24 h for Vero E6 cells (S1A), although there was a minor decrease in RMA observed with 30 µM of TAT-I24. For Calu-3 cells (S1B), the highest applied peptide concentration (30 µM of TAT-I24 and TAT) resulted in a significant decrease in RMA compared to the untreated control. Although cytotoxicity measurements indicated no severe effects of the peptides on the cell viability of Vero E6 cells, the highest applied concentration of peptides in the infection assays was 10 µM for both cell lines.

S2: Morphology of Vero E6 and Calu-3 cells after treatment with TAT-I24 or TAT after 24 h

Method:

In addition to the determination of the RMA, the cells were fixed with 4% formaldehyde after the measurement, as given in S1, and subsequently stained with crystal violet (CV) to investigate morphological alterations due to the presence of TAT and TAT-I24.

After fixation, the plates were washed three times with PBS and then incubated with 0.05% CV (in 20% methanol) and further diluted 1:70 in PBS for 30 min. The plates were washed with PBS another three times to remove the residues of CV and kept in a humid environment until microscopy (brightfield, 20x). The images were taken from representative areas of the wells.

Results:

Vero E6 cells displayed morphological alterations only in the presence of the highest concentration of TAT-I24 (30 µM), while TAT did not alter the cell morphology at any tested concentration (Figure S2A). The results from S1 and S2 suggested that the highest applicable peptide concentration without effects on Vero E6 cell shape was ≤10 µM, as was used in all experiments throughout the study and described in the main manuscript. For the Calu-3 cells, no alternations in the cell morphology were observed at any peptide concentration (Figure S2B), but as changes were detected in the RMA, the highest applied concentration was, the same as for the Vero E6 cells, 10 µM.

A

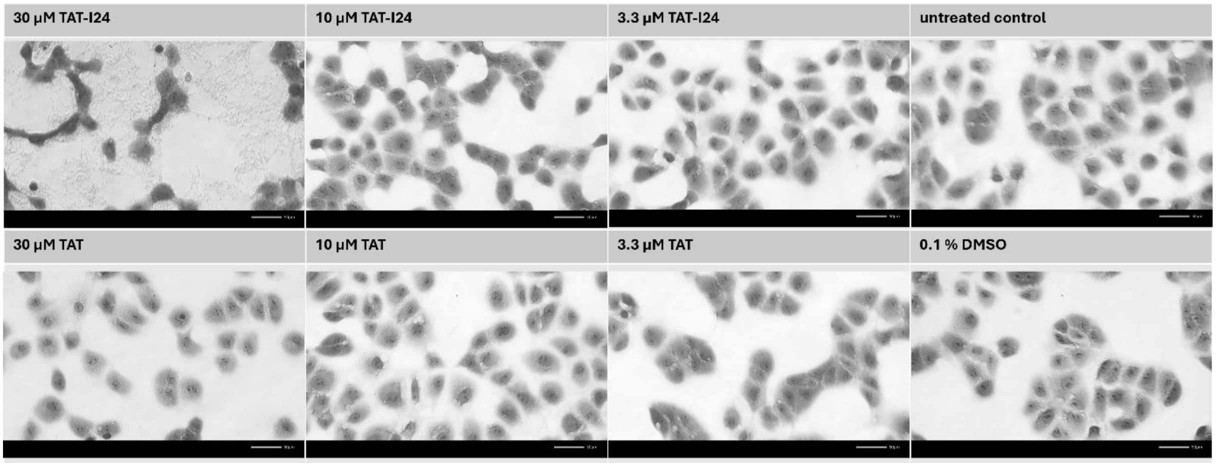

B

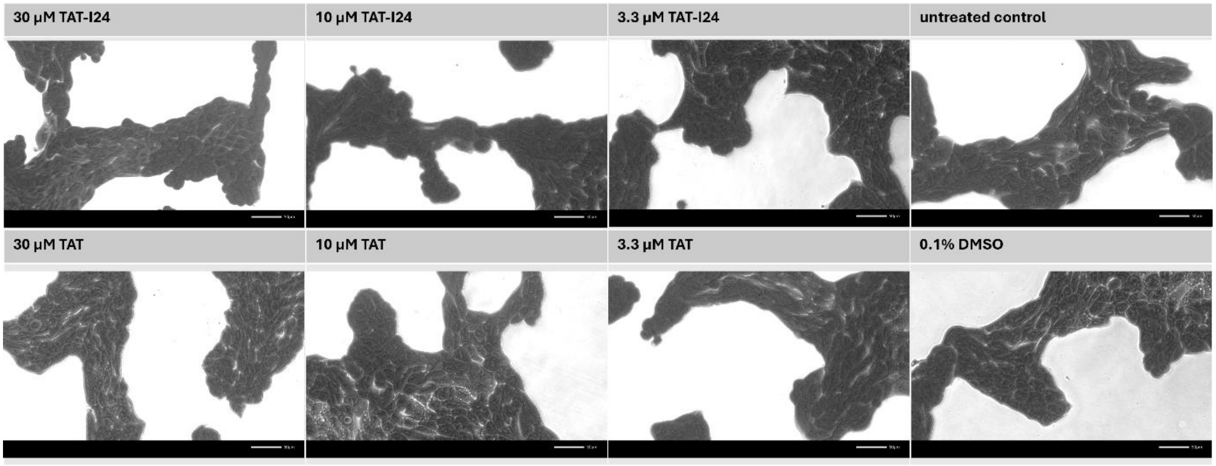

Figure S2. CV staining in Vero E6 and Calu-3 cells in the presence of peptides. Cell morphologies of Vero E6 cells (A) or Calu-3 cells (B) in the presence of increasing concentrations of TAT-I24 or TAT after 24 h treatment are shown. Scale bars indicate 50 µM. For imaging, a Nikon Eclipse TS100 microscope with 20-fold magnification was used along with a JENOPTIC GRYPHAX® camera and the corresponding software.

S3: SARS-CoV-2 nucleocapsid staining of Vero E6 cells treated with TAT-I24, TAT, or TAT-I24 analogs 24 h p.i. with the Wuhan variant

Method:

Infection and immunohistochemical staining were performed as described in Sections 2.4 and 2.6; the analysis of the acquired images was conducted as described in Section 2.13 of the main text.

Results:

In the presence of Remdesivir, TAT-I24, and the TAT-I24 analog C22del, the observed signal for SARS-CoV-2-nucleocapsid-positive stained cells was clearly reduced at 10 µM, while a partial reduction was observed with TAT and the analogs C14A, C20R, and C22M at 10 µM, and no or a minor reduction was observed with the peptide analogs C14P and C22del_cyc.

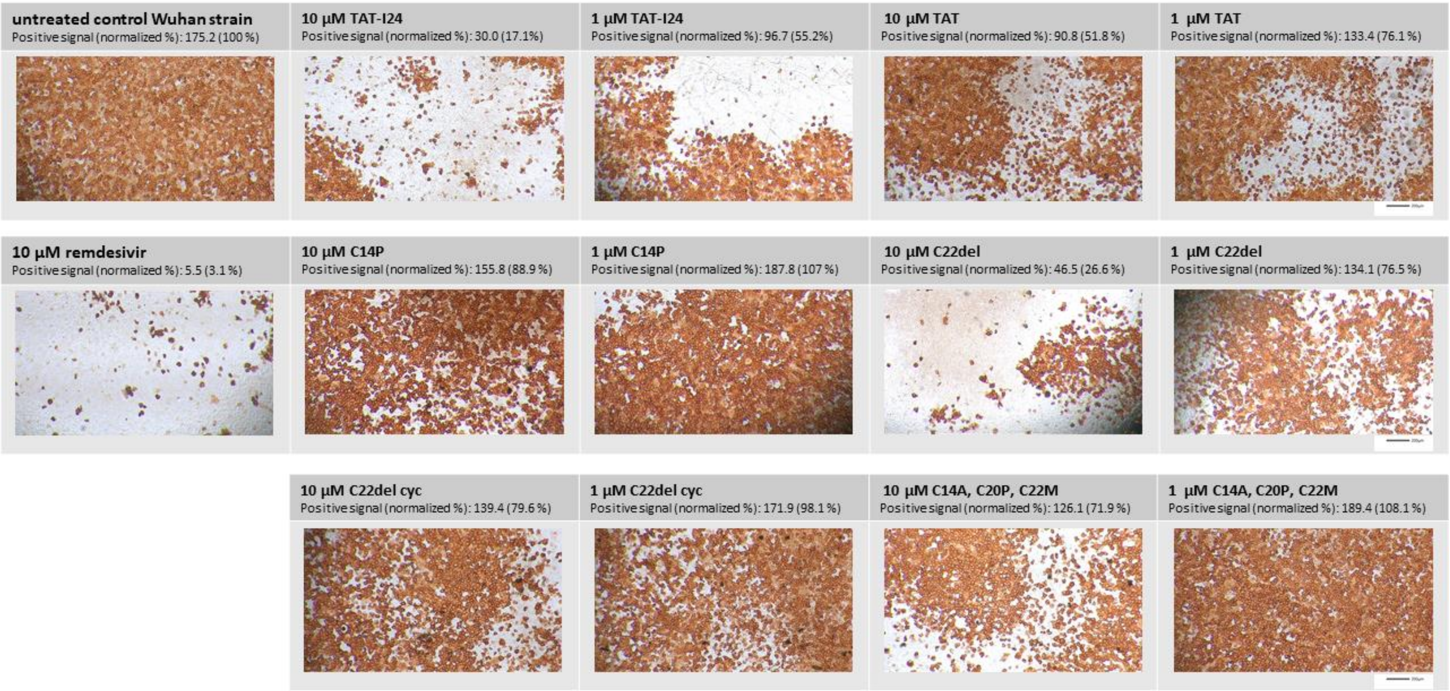

Figure S3: Immunohistochemical staining against the SARS-CoV-2 nucleocapsid in Vero E6 cells infected with the Wuhan variant in the presence of peptides. The images are the corresponding wells from which SARS-CoV-2 GE from the supernatants were determined and are shown in Figure 2A of the main text. The images were quantified for red-positive area signals and normalized to the untreated control; representative sections of the wells are shown. Scale bars indicate 200 µm.

S4: SARS-CoV-2 nucleocapsid staining of Vero E6 cells treated with TAT-I24, TAT, or scrambled peptides 24 h p.i. with the Wuhan variant

Method:

Infection and immunohistochemical staining were performed as described in Sections 2.4 and 2.6; the analysis of the acquired images was conducted as described in Section 2.13 of the main text.

Results:

In the presence of Remdesivir, TAT-I24, or TAT, SARS-CoV-2 nucleocapsid staining was reduced at 10 µM and 1 µM, while only a partial reduction was observed with scrambled peptide 1 at 10 µM and a minor reduction with scrambled peptide 2 at both concentrations.

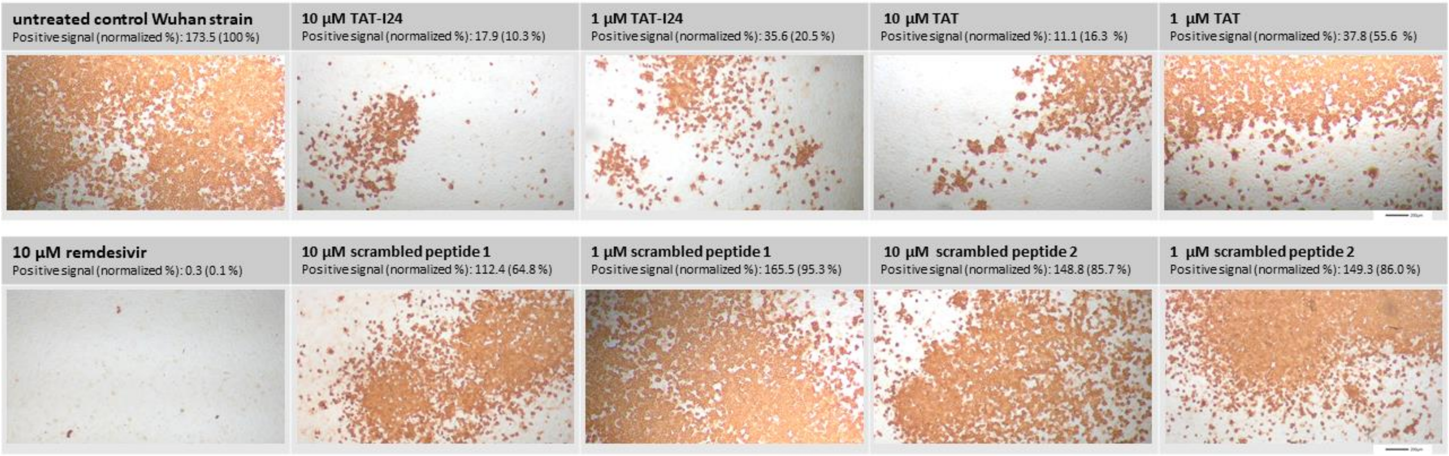

Figure S4: Immunohistochemical staining against the SARS-CoV-2 nucleocapsid in Vero E6 cells infected with the Wuhan variant in the presence of peptides. The images are the corresponding wells from which SARS-CoV-2 GE from the supernatants were determined and are shown in Figure 2B of the main text. The images were quantified for red-positive area signals and normalized to the untreated control; representative sections of the wells are shown. Scale bars indicate 200 µm.

S5: SARS-CoV-2 nucleocapsid staining of Vero E6 cells treated with CQ, TAT-I24, and a combination of the compounds 24 h p.i. with the Wuhan variant and the Delta variant

Method:

Infection and immunohistochemical staining were performed as described in Sections 2.4 and 2.6; the analysis of the acquired images was conducted as described in Section 2.13 of the main text.

Results:

Differential sensitivity of virus variants to CQ, TAT-I24, and a combination of both

In the presence of CQ, nucleocapsid staining (red-positive score) was dose-dependently inhibited when Vero E6 cells were infected with the Wuhan variant, while at concentrations below 0.1 μM of CQ, no inhibitory effect was observed, with a red-positive score rate comparable with the untreated control (Figure S5A). TAT-I24 alone reduced the red-positive signals by around 89%. A combination of CQ and 10 μM of TAT-I24 further enhanced the inhibitory effect, where only 2.1% of the red-positive signal compared to the untreated control was found in the presence of 3.3 μM of CQ + 10 μM of TAT-I24. However, the Delta variant revealed a different sensitivity profile (Figure S5B), with a less pronounced sensitivity at concentrations of CQ above 10 μM. Moreover, TAT-I24 did not cause any reduction in nucleocapsid staining when combined with any of the doses of CQ.

A

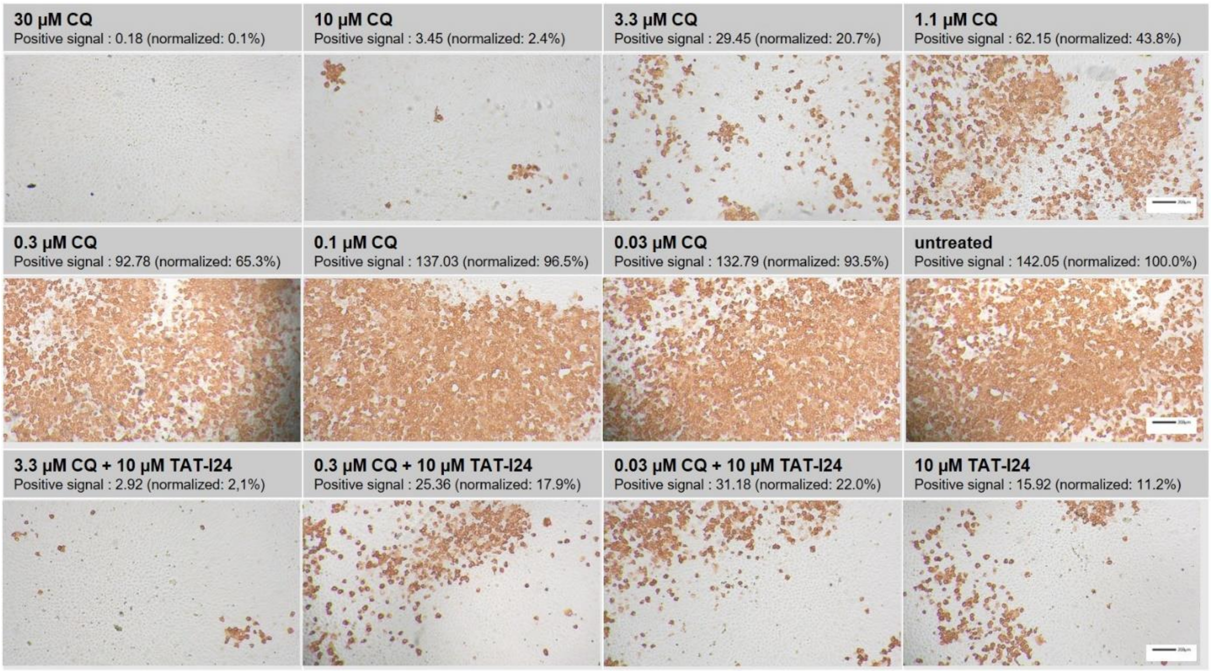

B

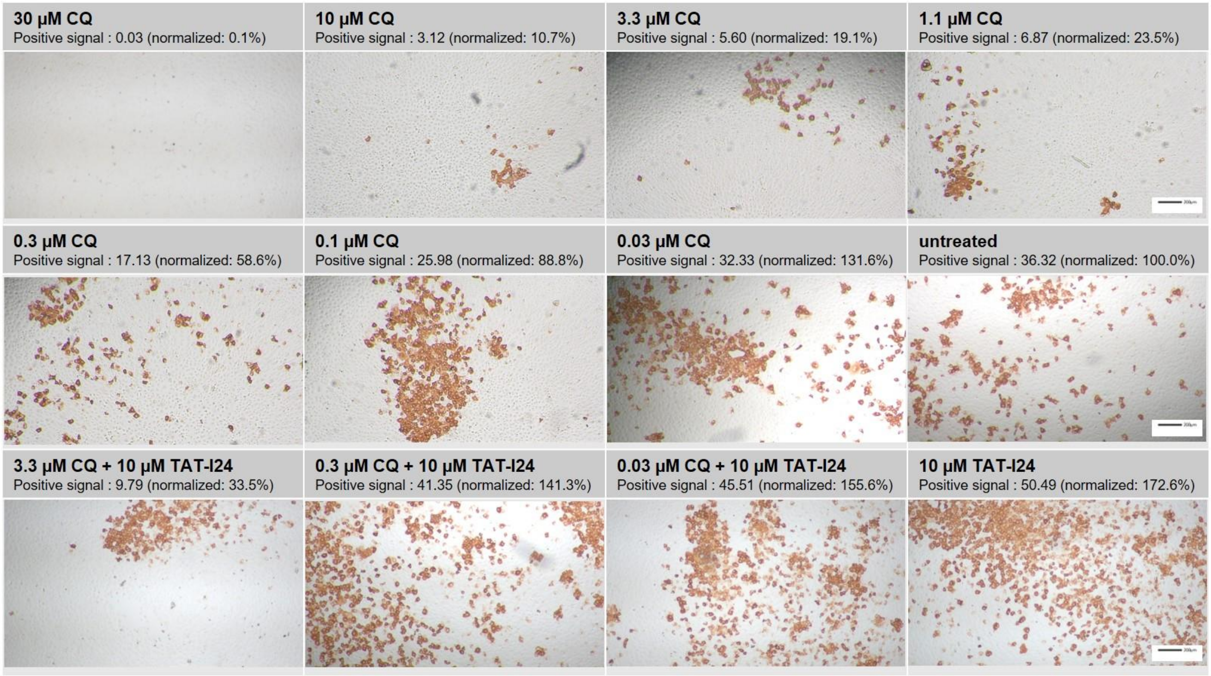

Figure S5: Immunohistochemical staining against the SARS-CoV-2 nucleocapsid in Vero E6 cells infected with the Wuhan variant (A) or the Delta variant (B). The images are corresponding wells from which SARS-CoV-2 GE from the supernatants were determined and are shown in Figure 5 of the main text. The images were quantified for red-positive area signals and normalized to the untreated control; representative sections of the wells are shown. Scale bars indicate 200 µm.
